# Supplementary material for: MetaRibo-Seq measures translation in microbiomes
Source: Nat Commun. 2020 Jun 29;11:3268. doi: 10.1038/s41467-020-17081-z (PMC7324362; doi:10.1038/s41467-020-17081-z)
Supplement: Supplementary file 10 — Supplementary Data 7 [file 41467_2020_17081_MOESM10_ESM.zip › File2/Confidence_High_Taxonomy/12551_out.krona.html]

Javascript must be enabled to view this page.

members
magnitude
magnitudeUnassigned
count
unassigned
taxon
rank

12551\_out

6

6
2
superkingdom

phylum
1239
6

class
91061
6

6
1385
order

90964
6
family

genus
6
1279

species

SRS024596\_contig\_number\_contig-100\_306.12416SRS047134\_contig\_number\_1273SRS075093\_contig\_number\_2159SRS077863\_contig\_number\_1826SRS077864\_contig\_number\_3667SRS102817\_contig\_number\_545
1282
6
